# The Use of Wearable Sensors in Human Movement Analysis in Non-Swimming Aquatic Activities: A Systematic Review

**DOI:** 10.3390/ijerph16245067

**Published:** 2019-12-12

**Authors:** Daniel A. Marinho, Henrique P. Neiva, Jorge E. Morais

**Affiliations:** 1 Department of Sport Sciences, University of Beira Interior, 6201-001 Covilhã, Portugal; henriquepn@gmail.com (H.P.N.); morais.jorgestrela@gmail.com (J.E.M.); 2 Research Center in Sports, Health and Human Development, CIDESD, 6201-001 Covilhã, Portugal

**Keywords:** human motion, aquatic, exercise, sensor

## Abstract

The use of smart technology, specifically inertial sensors (accelerometers, gyroscopes, and magnetometers), to analyze swimming kinematics is being reported in the literature. However, little is known about the usage/application of such sensors in other human aquatic exercises. As the sensors are getting smaller, less expensive, and simple to deal with (regarding data acquisition), one might consider that its application to a broader range of exercises should be a reality. The aim of this systematic review was to update the state of the art about the framework related to the use of sensors assessing human movement in an aquatic environment, besides swimming. The following databases were used: IEEE Xplore, Pubmed, Science Direct, Scopus, and Web of Science. Five articles published in indexed journals, aiming to assess human exercises/movements in the aquatic environment were reviewed. The data from the five articles was categorized and summarized based on the aim, purpose, participants, sensor’s specifications, body area and variables analyzed, and data analysis and statistics. The analyzed studies aimed to compare the movement/exercise kinematics between environments (i.e., dry land versus aquatic), and in some cases compared healthy to pathological participants. The use of sensors in a rehabilitation/hydrotherapy perspective may provide major advantages for therapists.

## 1. Introduction

The use of smart technology applied to the human movement/exercise analysis has been a must for researchers, clinicians, practitioners, and patients. Inertial measurement unit technology is a less time consuming, noninvasive, and practical alternative to the video-based methods used by researchers to analyze human motion, allowing to detect with a higher precision several parameters during exercise [1,2]. Technology advanced in a way that due to the sensor’ compactness (small size) multiple applications may be used in human locomotion, including exercise/sports movements [3,4].

Besides land movements, there are several physical activities that require physical movement in an aquatic environment. The use of sensors in water to evaluate some kind of human movement was extensively applied to swimming [5,6,7]. This activity being the most common sport performed in an aquatic environment, the main aims of such studies were the validation of accelerometers and the measurement of the specific kinematics related to swimming [6,8,9]. The validation process was dependent on two main factors inherent to the water environment such as the sealing and hypothetical drag caused by the sensor. Those studies evidenced that water-based exercises require the sensors to be hermetic sealed, making it water resistant/proof. Besides that, technology development also allowed to reduce the size of the sensor, which could be a great advantage for its use in a water environment due to drag [10]. Regarding the kinematic measurements, for an accurate acquisition, the sensor should be placed in a body segment that would not increase drag, would not bother the individual action, and would not limit the participant’s freedom of motion [11].

Nonetheless, human motion in the water is not only related to swimming. Walking and other movements/exercises (related to water-based sports activities and rehabilitation movements) are also human actions that are commonly analyzed in an aquatic environment. However, the use of sensors in such water-based activities in comparison to swimming is rather scarce [12,13]. Basically, such studies aimed to compare the walking pattern on dry land and in underwater conditions [12], propose movement analysis methodology based on inertial and magnetic sensors [14], assess the kinematics of underwater walking [13], perform some specific movements/exercises (e.g., squat and some variants) [15,16], compare abdominal pressure between dry land and underwater conditions [17], or monitor handicap people in hydrotherapy sessions [18]. The body of knowledge about the use and application of sensors technology is widely explored in a swimming perspective, with original studies and some systematic reviews. On the contrary, it seems that there is not a focused approach to its use in other exercises/movements performed in an aquatic environment. Hence, the aim of this study was to perform a systematic review of the studies related to the use of sensors assessing human movement in an aquatic environment, besides swimming.

## 2. Methods

### 2.1. Literature Search and Article Selection

The following databases were used to identify the studies that used wearables in an aquatic environment: IEEE Xplore, Pubmed, Science Direct, Scopus, and Web of Science. These electronic search databases were chosen as the most common databases related to methodological approaches in applied biomechanics in sports (performance, medicine, and engineering). The inclusion criteria were set as (i) written in English; (ii) published in a peer-reviewed journal; (iii) related to the analysis of human movement in an aquatic environment (excluding swimming), performed at a maximum of 2 m of depth. Review papers, conference papers and books, studies including animals, and publications not related to the topic in question were excluded from the analysis.

**Figure 1:**
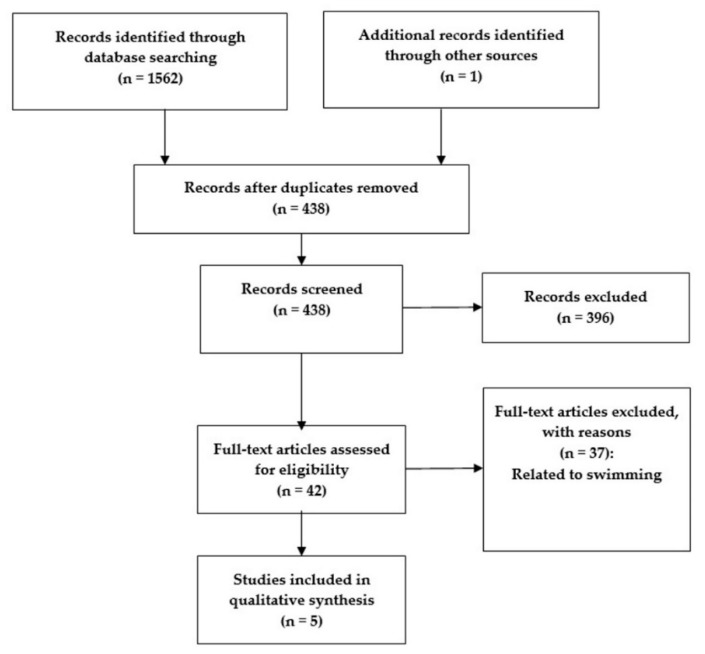
Flow diagram representing the different phases of paper selection for the systematic review.

The keywords and/or combinations used for the search were in-water, aquatic, wearable, sensor, accelerometer, gyroscope. As an initial search strategy, the title, abstract, and keyword fields of the text were first identified and read carefully for a first selection of the journal articles. If one of these fields (title, abstract, and keywords), was not clear about the topic in analysis, the complete article was read and fully reviewed to ensure its inclusion. After deleting all unrelated and duplicated articles (and also excluding all articles related to swimming), five articles were included in the final review published until June 2018 (Figure 1). From such articles, the reviewers extracted information about the purpose of the study, the participants, characteristics of the sensor(s) used, the body area where the sensor was allocated, the variables measured, and the data analysis used.

### 2.2. Quality Assessment

The PEDro scale was used to assess the quality of the articles selected. It was observed that this approach (i.e., PEDro scale) is an indicator of the methodological quality. Two independent reviewers fully read all the included articles and scored according to the items of the scale (poor quality if scored ≤3; fair quality if scored 4–5; high quality if scored 6–10) [19]. Afterward, Cohen’s Kappa (K) was computed to assess the agreement between reviewers. It was interpreted as (i) no agreement if K ≤ 0; (ii) none to slight if 0.01 < K ≤ 0.20; (iii) fair if 0.21 < K ≤ 0.40; (iv) moderate if 0.41 < K ≤ 0.60; (v) substantial if 0.61 < K ≤ 0.80; (vi) almost perfect if 0.81 < K ≤ 1.00 [20].

After reviewing all articles included, the PEDro scale showed a mean score of 5.25 ± 0.61 (i.e., fair quality), and Cohen’s Kappa an almost perfect agreement between reviewers (K = 0.96, *p* < 0.001).

## 3. Results

Table 1 present the research studies included in the analysis, and the summary of each one regarding the aim, participants, and a set of inherent specifications of the sensors used. 

### 3.1. Sensor Specifications

Three studies reported the utilization of eight sensors, characterized by accelerometry, gyroscope, and magnetometer features and with an acquisition frequency of 128 Hz [12,13,14]. One study provided more information about the sensors used such as size and weight [13]. Moreover, the authors provided information about the three kinds of usage that each sensor has: (i) accelerometer (range: ±2 g, ±6 g; bandwidth: 50 Hz; resolution: 14 bit; noise: $128\mathrm{ug}/\sqrt{\mathrm{Hz}}$); (ii) gyroscope: (range: ± 2000°/s; bandwidth: 50 Hz; resolution: 14 bit; noise: 0.07°/s/$\sqrt{\mathrm{Hz}}$); (iii) magnetometer: (range: ±6 Gauss; bandwidth: 50 Hz; resolution: 14 bit; noise: $4\;m\;Gauss/\sqrt{\mathrm{Hz}}$). Two other studies used five sensors (tri-axial accelerometer and gyroscope) with an acquisition frequency of 100 Hz [15,16]. None of the four selected studies report any kind of information about the sensors’ sealing.

### 3.2. Participants

The studies analyzed reported the number of male and female participants, their age, height, and body mass. The study by Fantozzi et al. [12] reported that the participants were healthy (males and females), and Severin et al. [16] reported such data per sex. Other studies split the participants into healthy or with some pathology (in that study, particular cases had chronic anterior knee pain) and did not mention the participants’ sex [15]. It only mentioned that both groups (young adults with chronic anterior knee pain and healthy adults) were age and sex matched. The study by Mangia et al. [13] also split the participants in young healthy adults (males and females pooled together), healthy elderly (males and females pooled together), and one pathological participant. Only one pathological participant (anterior cruciate ligament injury) was included in the study conducted by Cortesi et al. [14].

### 3.3. Anatomical Allocation of the Sensor

Table 2 presents the body area where the sensor was allocated, and also the variables computed and the data analysis and statistics used. Three studies used eight sensors and allocated them in the thorax (one sensor), pelvis (one sensor), thighs (two sensors), shanks (two sensors), and feet (two sensors) [12,13,14]. The remaining studies [15,16] used five sensors and allocated them in the thorax (one over the spinous process of the third thoracic vertebra), in the thighs (two attached bilaterally to the participant’s lateral mid-thigh), and in the shanks (two attached bilaterally to the participant’s lateral shank).

### 3.4. Monitoring Applications

All four studies monitored the parameters related to the lower limbs. The studies conducted by Fantozzi et al. [12] and Cortesi et al. [14] analyzed the kinematics of the thorax-pelvis and lower limb joints, in sagittal and frontal planes, during walking in an underwater condition (especially the movements of flexion-extension). Another study monitored the trunk and lower limbs as a multibody joint during underwater walking [13]. Parameters such as flexion-extension, tilting, and rotation were analyzed. The two remaining studies [15,16] analyzed the difference (in the inclination angles of the trunk, thighs, and shanks) between specific movements that can be performed in water: squat, split squat, and single-leg squat. In one study, the authors quantified the differences between movements [16], and in the other study the authors assessed the asymmetries between healthy participants and participants with chronic anterior knee pain [15]. Table 2 presents in detail all variables analyzed in each study, and Table 3 the summary of the main results of each study. 

## 4. Discussion

The aim of the current study was to review and summarize the existing studies using sensor technology in aquatic environment movements/exercises, besides swimming. The number of studies applying such sensors in an aquatic environment, in non-swimming exercises/activities, revealed to be limited. Overall, the studies selected aimed to assess the underwater walking kinematics [12,13,14], or a set of specific squat exercises performed in an aquatic environment [15,16].

### 4.1. Sensor Specifications and Sealing

Overall, the studies included revealed information about the number of sensors used, and the sensor type (tri-axial accelerometer and gyroscope, with two studies adding also a magnetometer characteristic). Another characteristic revealed was the frequency acquisition. The studies related to underwater walking used a 128 Hz sensor, and the studies assessing the squat exercise a 100 Hz sensor. The study conducted by Mangia et al. [13] did present more detailed information about the sensors’ characteristics (Table 1). Regarding the sealing of the sensors, three studies referred to it [12,13,14]. The authors from both studies reported that the sensors were inserted in round plastic waterproofed boxes and fixed with elastic bands to the body segments for analysis. One might consider that such short information about the sensor’s specifications and sealing happens because that information is already provided in the existing literature about this topic in swimming (since the sensor characteristics should be similar, as the environment also is).

Indeed, swimming is the most popular and interventional human activity in the aquatic environment, where studies aimed to validate the use of sensors and measure several parameters related to swimming [21,22,23]. At least two studies [11,24] reviewed the use of sensor technology in swimming, and gathered the characteristics of the sensors used. Moreover, it was suggested that the sealing is an important factor to take into account, as the data logging and transmission might be violated [11]. Nevertheless, it was expected that such information would have been deeply explored in the selected studies about human locomotion/movement in an aquatic environment.

### 4.2. Participants, Purpose, and Anatomical Allocation of the Sensor

The samples included in all the studies comprised in this systematic analysis, gathered both male and female participants aiming to (i) compare a young adult group (males and females) to a pathological (anterior knee pain) sex and age matched group performing a double-leg squat and a single-leg squat on land and in water [15]; (ii) quantify the kinematical differences performing mixed types of squats on land versus in water of healthy university students [16]; (iii) estimate 3D joint kinematics (lower limbs) based in the underwater walking of healthy adults participants, and compare it to dry land walking [12]; (iv) propose a movement analysis methodology based on inertial and magnetic sensors to provide quantitative data on the joint kinematics of a pathological participant (anterior cruciate ligament injury) [14]; (v) validate a sensor (inertial-magnetic measurement unit) for in water walking, comparing it to dry land walking, in healthy young adults, healthy elderly, and in a pathological male (anterior cruciate ligament) [13].

Other studies that were not included in this systematic research (due to inclusion criteria), compared the abdominal pressure performing specific drills in water and on land [17], developing a smart suit for monitoring in water body kinematics activities in a hydrotherapy approach [25,26,27], and monitoring handicapped people in an aquatic environment [18]. Hence, it can be summarized that the studies published about human movement in an aquatic environment using sensors, especially the ones included in this analysis, aimed to analyze the lower limbs’ kinematics (walking and performing a set of squat exercises) of healthy young adults, healthy elderly, and participants with specific pathologies affecting their lower limbs. By such fact, the sensors were allocated in the thorax and lower trunk (pelvis, thighs, and shanks). Moreover, it might be suggested that the holistic perspective of such studies is in a hydrotherapy base.

### 4.3. Monitoring Applications

Besides swimming and head-out aquatic exercises (where the upper limbs are also responsible for some actions) [28], all remaining movements/exercises simulate patterns that are similar and/or are performed equally in a dry land environment [29,30]. Overall, the data gathered from this analysis revealed that all studies analyzed movements based on lower limb kinematics (i.e., actions responsible for displacement: walking; and for the exercise: squats). Fantozzi et al. [12] compared the dry land and underwater walking in healthy participants, and Mangia et al. [13] in healthy young adults, healthy elderly, and a pathological participant. Overall, both studies aimed to measure joint angles and gait kinematics (Table 2). Both studies showed a 40% decrease in the underwater walking compared to dry land, a shorter stride length and a higher stride duration in underwater conditions. Indeed, it was indicated that such differences were mainly related to the water drag force during movement, lower apparent body weight in water, and lower comfortable walking speed the participants selected in water [31]. Concerning the joint angles analyzed (Table 2), all angles were higher in the underwater condition compared to the dry land, except the knee flexion-extension range of motion (dry land: 64.9 ± 3.8°; underwater: 60.0 ± 18.0°) [12].

The study by Mangia et al. [13] found similar results for healthy young adults. As for the healthy elderly group, they presented higher stride duration in comparison to the younger group (5% in percentage of stride cycle), and a stride length (49%) and walking speed (54%) significantly lower. This elderly group also presented flexed kinematics regarding the knee at the heel strike (−8°) and a more dorsiflexed ankle (−8°). Overall, the elderly group showed higher values of flexion at all joints analyzed [13]. The difference found in knee and hip flexion-extension between the elderly and younger groups could be explained by the effect of the walking speed in the elderly participants (i.e., lower walking speed). Additionally, the kinematic analysis showed an increase of the knee and hip flexion and a decrease of the plantar flexion in elderly participants in comparison to the younger group. Such differences could be associated with (i) a subtle hip flexion contracture, and (ii) an ankle plantar flexor concentric weakness of the elderly participants [13]. Concerning the pathological participant, the data presented in the study conducted by Mangia et al. [13] is a summary of the one presented in Cortesi et al. [14]. The authors showed meaningful differences in the gait pattern of an injured participant (anterior cruciate ligament injury) when comparing the injured side to the contralateral one. The injured limb presented a reduction in the maximum flexion and in the range of motion when comparing it to the young adult group (56 to 36°, and 60 to 39°, respectively). Moreover, the overall knee range of motion and the hip range of motion of the injured limb were significantly lower in comparison to the contralateral side (47% and 64% of the contralateral one, respectively).

Other studies aimed to monitor the kinematic pattern of several squat exercises [15,16]. One assessed the asymmetries between a young healthy group and an age- and sex-matched group with anterior knee pain (in dry land vs. aquatic environment) [15], and the other assessed the differences within a young healthy group (university students), comparing the exercise performed in dry land and in an aquatic environment [16]. Regarding the first study, significant differences were found between limbs while performing the exercise in underwater conditions for the thigh lateral deviation, knee adduction, and hip abduction. Furthermore, significant differences (with a moderate-large effect) were found in the affected limb when comparing the double squat in dry land (shank medial deviation: 8.2 ± 5.7°; thigh lateral deviation: 13.6 ± 9.4°; knee adduction: 20.0 ± 13.7°; hip abduction: 12.7 ± 9.9°) with underwater conditions (shank medial deviation: 11.9 ± 4.2°; thigh lateral deviation: 20.6 ± 9.0°; knee adduction: 30.3 ± 11.6°; hip abduction −18.8 ± 10.7°).

The same trend was observed for the single-leg squat. The affected limb presented significant (with moderate-large effect) differences when comparing the drill in dry land versus underwater conditions, in all variables analyzed (except the thorax lateral deviation, hip adduction, and knee abduction. In the second study [16], the authors showed that water immersion (until the great trochanter) did not limit the range of depth during the squat and split squat. It also allowed the participants to achieve higher depth during the single-leg squat. In the sagittal and frontal plane displacement, the differences found in all three types of squats between dry land and underwater, for the three segments analyzed (thorax, thigh, and shank), were higher in the early ascent and in the late descent phases of the movement. Overall, the coefficient of variation presented moderate-large effects on the segment’s movement variability (sagittal and frontal planes), being larger in the underwater condition (especially for the split squat and single squat-shank and thigh; and for the squat-thorax).

## 5. Conclusions

Summarizing the exercises and/or movements assessed in an aquatic environment throughout the use of sensors, walking and squat exercises were those mainly chosen. Both showed differences between environments. Overall, the exercises/movements performed in the aquatic environment were different in comparison to dry land (especially when comparing to injured participants with anterior knee injury). In a walking perspective, the studies analyzed presented similar results to the ones reported in studies using a video-based approach [31], but with the advantage of allowing a higher number of steps, and hence a walking rhythm more similar to the one in a daily basis [12]. Walking is characterized by a cyclic pattern, where external constrictions may induce internal (movement) restrictions. For example, asking a participant to walk during a small amount of time (to be filmed by a camera), may constrict his/her movement pattern, especially in a non-native environment (aquatic). Hence, the possibility to acquire several cycling gaits (allowing the participant to stabilize his/her pattern), is a must for researchers, also allowing analyzing a more realistic simulation of a movement generally performed on dry land. On the other hand, performing the exercises/movements (squat and other variants) presented in the studies gathered for analysis, the participants are placed in the same spot. Therefore, the video analysis is not as restricted as it can be for walking. Nevertheless, analyzing several repetitions of such specific exercises/movements in a video-based approach is not as immediate as using smart technology (sensors). This is one of the major mainstreams of using sensors, i.e., the data is immediately disposed for analysis.

Overall, it might be concluded that the use of sensors to measure human exercises/movements in an aquatic environment aimed to compare the movement/exercise kinematics between environments (i.e., dry land versus aquatic), and in some cases comparing healthy to pathological participants. Differences were found between the gait or squat kinematics when walking or performing the exercise in different environments. Understanding such differences might be a major outcome in a rehabilitation/hydrotherapy point of view. The water properties (hydrostatic pressure, buoyancy, viscosity, and thermodynamics) combined with the biomechanical effects of water immersion present several advantages to the human body while performing movements/exercises in an aquatic environment. Thus, such rehabilitation programs and/or hydrotherapy sessions may have major advantages by measuring the movement/exercise in a sensor approach base.

The following major advantages were highlighted: (i) simple and fast set-up; (ii) practical calibration; (iii) less time-consuming processing; (iv) a wider field of acquisition allowing to record and analyze a higher number of strides/strokes (video-cameras limit the field of data acquisition). On the other hand, these studies did point out some cautions and/or advices when using this technology: (i) wearable sensors could be less accurate (i.e., higher measurement error) in analyzing the joint kinematics of the lower limbs in the transverse plane (but such difference is not meaningful); (ii) wireless communication might not work in an aquatic environment, so the data cannot be analyzed in real-time; (iii) there could be a risk of slight discrepancies in the sensor allocation between testing sessions and participants.

## Figures and Tables

**Table 1:** List of the articles selected for analysis, including the article aim and sample, as well as the sensor specifications.

Source	Aim	Participants	Sensor Specifications					
			Units	Type	Sizes	Weight	Frequency	Sealing
Fantozzi et al. [12]	To estimate the 3D joint kinematics of the lower limbs and thorax-pelvis joints in sagittal and frontal planes during underwater walking using wearable inertial and magnetic sensors (comparing to dry land).	11 healthy participants (6 males and 5 females: 27.0 ± 3.4 years; 174.2 ± 8.2 cm of height; 70.2 ± 11.8 kg of weight).	8	Accelerometer gyroscope magnetometer	Not described	Not described	128 Hz	Inserted in a round plastic waterproofed box
Mangia et al. [13]	Instrumental validation of inertial-magnetic measurements units (IMMUs) in water, and the description of their use in clinical and sports aquatic applications applying customized 3D multibody models	11 healthy young adults (6 males and 5 females: 27.0 ± 3.4 years; 174.2 ± 8.2 cm of height; 70.2 ± 11.8 kg of mass). Healthy elderly (3 males and 2 females: 71.6 ± 2.2 years; 167.8 ± 6.9 cm of height; 67.0 ± 13.0 kg of mass). One pathological male (left anterior cruciate ligament injury; 39 years; 171 cm of height; 85 kg of mass)	8	Accelerometer (3 axes) gyroscope (3 axes) magnetometer (3 axes)	48.4 × 36.5 × 13.4 mm	<22 g	128 Hz	inserted in a round plastic waterproofed box
Cortesi et al. [14]	Propose a movement analysis methodology based on inertial and magnetic sensors to provide quantitative data on the joint kinematics of an anterior cruciate ligament injured patient	One pathological male (left anterior cruciate ligament injury; 39 years; 171 cm of height; 85 kg of mass)		Accelerometer gyroscope magnetometer	Not described	Not described	128 Hz	Inserted in a round plastic waterproofed box
Severin et al. [15]	To assess bilateral kinematics during double-leg squats and single-leg squats on land and in water in individuals with unilateral anterior knee pain. Additionally, to quantify bilateral asymmetry in both environments in affected and unaffected individuals using a symmetry index	20 young adults with chronic anterior knee pain (10 males and 10 females), and 20 healthy age- and gender-matched adults (anterior knee pain group: 22.8 ± 4.0 years, 71.2 ± 13.0 kg of body mass, 1.72 ± 0.09 m of height; control group: 22.2 ± 2.9 years, 67.6 ± 13.4 kg of body mass, 1.72 ± 0.10 m of height)	5	Tri-axial accelerometers and gyroscopes	Not described	Not described	100 Hz	Not described
Severin et al. [16]	To use inertial sensors to quantify differences in kinematics and movement variability of bodyweight squats, split squats, and single-leg squats performed on dry land and whilst immersed to the level of the greater trochanter	25 active healthy university students (11 females: 21.6 ± 2.3 years, 1.64 ± 0.06 m of height, 59.2 ± 10.3 kg of body mass; 14 males: 22.6 ± 3.3 years, 1.77 ± 0.08 m of height, 75.3 ± 10.5 kg of body mass)	5	Tri-axial accelerometers and gyroscopes	Not described	Not described	100 Hz	Not described

**Table 2:** Summary of the body area and variables assessed, and the data analysis and statistics performed.

Source	Body Area	Allocation Indications	Variables	Data Analysis and Statistics
Fantozzi et al. [12]	Thorax (1), pelvis (1), thighs (2), shanks (2), feet (2).	The sensor on the thorax was placed in the middle area between the incisura jugularis and processus xiphoideus. The sensor on the pelvis should be placed with the x-Opal-axis aligned with the left-right axes line. The sensors on the thighs were placed in the central-third, with the z-Opal-axis pointing laterally. The sensors on the shanks were placed slightly above the lateral malleolus, with the z-Opal-axis pointing perpendicular to the sagittal plane. The sensors on the feet were placed over the flat portion of the lateral part of the metatarsal area.	Stride duration, stance percentage, stride distance, flexion-extension at toe-off, flexion-extension maximum, flexion-extension minimum, flexion-extension at heel strike, flexion-extension maximum, flexion-extension range of motion, dorsi-plantar flexion at heel strike, dorsi-plantar flexion range of motion, inversion-eversion at toe-off, inversion-eversion mean.	Linear mixed models were applied to identify the effects of the environment (land or water) and walking speed, and their interaction with each variable.
Mangia et al. [13]	Thorax, pelvis, thighs (2), shanks (2), feet (2).	The sensor on the thorax was placed in the middle area between the incisura jugularis and processus xiphoideus, aligning the x-sensor axis to the long axis of the sternum. The sensor on the pelvis was placed with the x-sensor axis aligned with the left-right axes line. The sensors on the thighs were placed in the central-third, with the z-sensor axis pointing laterally. The sensors on the shanks were placed slightly above the lateral malleolus, with the z-sensor axis pointing perpendicular to the sagittal plane. The sensors on the feet were placed over the flat portion of the lateral part of the metatarsal area.	Thorax-pelvis joint (posterior-anterior tilting, right drop-rise, right internal-external rotation), hip and knee joints (flexion-extension, abduction-adduction, internal-external rotation), ankle joint (dorsi-plantar flexion, ankle inversion-eversion, internal-external rotation).	One-way nonparametric ANOVA test to evaluate significant differences between groups (young adult vs. elderly vs. pathological patients).
Cortesi et al. [14]	Thorax (1), pelvis (1), thighs (2), shanks (2), feet (2).	The sensor on the thorax was placed in the middle area between the incisura jugularis and processus xiphoideus. The sensor on the pelvis should be placed with the x-Opal-axis aligned with the left-right axes line. The sensors on the thighs were placed in the central-third, with the z-Opal-axis pointing laterally. The sensors on the shanks were placed slightly above the lateral malleolus, with the z-Opal-axis pointing perpendicular to the sagittal plane. The sensors on the feet were placed over the flat portion of the lateral part of the metatarsal area.	Stride duration, stance percentage, stride distance, walking speed, hip flexion-extension, knee flexion extension, knee flexion-extension at heel strike, knee flexion-extension at toe-off, maximum knee flexion-extension, gait cycle percentage at maximum knee flexion-extension, ankle dorsi-plantar flexion, gait cycle percentage at minimum ankle dorsi-plantar flexion.	Matlab was used to process the data, computing the lower limbs 3D joint angles and segmented cycle gaits. Means and standard deviations were computed.
Severin et al. [15]	Thorax (1), lateral mid-thigh (2) and shank (2).	Sensors were placed bilaterally halfway between the proximal and distal joint centers of the thighs and shanks. One sensor was positioned over the third thoracic vertebra and another was attached to the sacrum.	Inclination of the thorax, thigh, and shank segments between land and aquatic based movements (double-leg squat and single-leg squat).	Mean differences to assess asymmetries between limbs, and covariance to determine differences between environments (land vs. water).
Severin et al. [16]	Thorax (1), lateral mid-thigh (2) and shank (2).	Sensors were attached bilaterally to the participant’s lateral mid-thigh and shank, halfway between the proximal and distal joint centers. One sensor was positioned over the spinous process of the third thoracic vertebra. The allocation of the sensors was measured to be at equal distance from the proximal and distal joint centers for the lower body segments to ensure consistency. For the squat depth, one additional sensor was attached to the sacrum, at equal distance from the posterior superior iliac spines.	Degree angle of the thorax, thigh, and shank segments, performing the squat, split squat, and single-leg squat.	Mean differences between environments (land vs. water) and coefficient of variations to assess the variability of the individual waveforms.

**Table 3:** Summary of the main results of each study included in the analysis.

Source	Main Results
Fantozzi et al. [12]	Walking speed in underwater environment was 40% slower in comparison to dry land, the stride length being shorter. Similar patterns in the joint angle were observed. However, during the heel strike a more dorsiflexed ankle and a more flexed knee were observed in the underwater condition. The hip showed the difference during the last phase of the stance, reporting a higher flexion at toe-off. The joint angles patterns of the thorax-pelvis and of the hip in the frontal plane were smoother in the underwater environment (due to the speed reduction).
Mangia et al. [13]	Walking in an aquatic environment showed a reduction of median speed, longer stride duration, and shorter stride distance in comparison to dry land. Differences were found in flexion-extension of the knee and ankle at heal strike, and of the hip at toe-off between underwater and dry land environments. Elderly participants showed an increased median stance duration percentage with respect to that of young adults and a decreased median swing duration and duration percentage. No differences were found in the spatiotemporal analysis between the injured and the contralateral sides in pathological participants. Nonetheless, as different joint kinematic variables were found, the authors suggested using 3D joint kinematics variables to have a deeper understanding of the patient biomechanics.
Cortesi et al. [14]	Walking in underwater environment increases the flexion-extension range of motion of the injured limb being more similar to the one presented by the control group with respect to dry land walking. In this sense, patients will assume gait patterns more similar to those of the control group from the temporal gait events perspective. Moreover, it was highlighted that the increment of the knee flexion-extension range of motion should be one of the first functionalities to be restored in a patient with anterior cruciate ligament injury after surgery. Hence, aquatic therapy seems to provide beneficial effects in this direction.
Severin et al. [15]	Participants with anterior knee pain presented different kinematics in the affected and unaffected limbs during double-leg squat and single-leg squat performed in an aquatic and dry land environment. The water immersion (reduced load) allowed individuals with anterior knee pain to achieve greater squat depth during both double- and single-leg squat in comparison to when performing the exercises on dry land. Compensatory movements presented by the anterior knee pain group on land may therefore aggravate their condition further. Nonetheless, this adaptation was not reduced in water (despite the reduced load). Overall, the increased range of motion performed in an aquatic environment led to early rehabilitation goals in anterior knee pain patients.
Severin et al. [16]	Water immersion at the greater trochanter level did not limit the depths of squats and single squats, allowing participants to maintain a range of movement similar to the one presented in the dry land environment. This was even enhanced for the single-leg squat (higher depth in water). Gravitational offloading and viscosity inherent to the aquatic environment reduced the participants’ reliance on their body position for stability allowing them to use a more upright trunk posture. Hence, the aquatic environment encourages more vertically aligned trunk and shank segments with an overall smaller range of motion, and increased movement variability. Squats performed in an aquatic environment are indicated for lower body rehabilitation as water immersion emphasizes improved technique without changing the overall movement pattern.
